# The Role of Tau beyond Alzheimer’s Disease: A Narrative Review

**DOI:** 10.3390/biomedicines10040760

**Published:** 2022-03-24

**Authors:** Eleonora Virgilio, Fabiola De Marchi, Elena Contaldi, Umberto Dianzani, Roberto Cantello, Letizia Mazzini, Cristoforo Comi

**Affiliations:** 1 MS Centre, Department of Neurology, Maggiore della Carità Hospital, University of Piemonte Orientale, 28100 Novara, Italy; virgilioeleonora88@gmail.com (E.V.); roberto.cantello@med.uniupo.it (R.C.); 2 Ph.D. Program in Medical Sciences and Biotechnologies, Department of Translational Medicine, University of Piemonte Orientale, 28100 Novara, Italy; contaldie@yahoo.it; 3 Neurology Unit, S. Andrea Hospital, Department of Translational Medicine, University of Piemonte Orientale, 13100 Vercelli, Italy; cristoforo.comi@med.uniupo.it; 4 ALS Centre, Department of Neurology, Maggiore della Carità Hospital, University of Piemonte Orientale, 28100 Novara, Italy; mazzini.letizia5@gmail.com; 5 Movement Disorders Centre, Neurology Unit, University of Piemonte Orientale, 28100 Novara, Italy; 6 Clinical Biochemistry, Department of Health Sciences, University of Piemonte Orientale, 28100 Novara, Italy; umberto.dianzani@med.uniupo.it

**Keywords:** Tau, neurodegeneration, biomarkers, multiple sclerosis, amyotrophic lateral sclerosis, frontotemporal spectrum disorder, Parkinson’s disease, tauopathies, prognosis

## Abstract

Nowadays, there is a need for reliable fluid biomarkers to improve differential diagnosis, prognosis, and the prediction of treatment response, particularly in the management of neurogenerative diseases that display an extreme variability in clinical phenotypes. In recent years, Tau protein has been progressively recognized as a valuable neuronal biomarker in several neurological conditions, not only Alzheimer’s disease (AD). Cerebrospinal fluid and serum Tau have been extensively investigated in several neurodegenerative disorders, from classically defined proteinopathy, e.g., amyotrophic lateral sclerosis (ALS), frontotemporal dementia (FTD), and Parkinson’s disease (PD), but also in inflammatory conditions such as multiple sclerosis (MS), as a marker of axonal damage. In MS, total Tau (t-Tau) may represent, along with other proteins, a marker with diagnostic and prognostic value. In ALS, t-Tau and, mainly, the phosphorylated-Tau/t-Tau ratio alone or integrated with transactive DNA binding protein of ~43 kDa (TDP-43), may represent a tool for both diagnosis and differential diagnosis of other motoneuron diseases or tauopathies. Evidence indicated the crucial role of the Tau protein in the pathogenesis of PD and other parkinsonian disorders. This narrative review summarizes current knowledge regarding non-AD neurodegenerative diseases and the Tau protein.

## 1. Introduction

In neurological disease management, there is a considerable demand for reliable fluid biomarkers to improve differential diagnosis and for prognostic purposes and the prediction of treatment response. Additionally, the presence of neurodegenerative processes in neurological diseases could be determined or rejected by specific fluid biomarkers and, therefore, helpful for subsequent clinical management. The Tau protein, along with beta-amyloid (Aβ), represents a milestone in Alzheimer’s disease (AD) diagnosis [1]. However, with Tau being a microtubular protein that reflects axonal loss, in recent years, evidence has been collected, particularly in cerebrospinal fluid (CSF) of multiple sclerosis (MS) subjects, to examine its role as a diagnostic and prognostic biomarker. Moreover, a pathological hyperphosphorylated form of the Tau protein (p-Tau) may be released during neurodegenerative processes, leading to a high volume of evidence supporting that total Tau (t-Tau), p-Tau, and its ratio, may be useful in amyotrophic lateral sclerosis (ALS) and frontotemporal spectrum disorder (FTSD) differential diagnosis. Finally, a crucial role of Tau in the pathogenesis of Parkinson’s disease (PD) and other parkinsonian disorders has been unveiled, leading to exciting future perspectives. This narrative review focused on summarizing the Tau protein’s role as a biomarker beyond AD disease. We searched studies published in worldwide, well established scientific databases, mainly PubMed/Medline.

## 2. Structure, Function, and Measurement of the Tau Protein 

**Figure 1:**
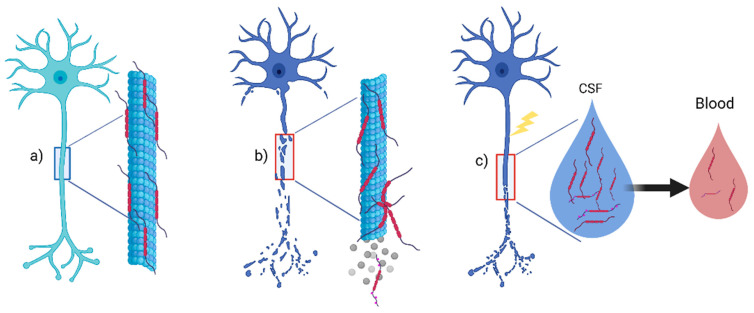
Tau protein in the central nervous system. (**a**) Tau protein is a microtubule associated protein (MAP) that contributes with others MAP to axonal stabilization in healthy neurons; (**b**) phosphorylation of Tau will reduce affinity for microtubule, and in many neurodegenerative diseases, hyperphosphorylated-Tau will induce neuronal death; (**c**) upon any axonal damage from aging or pathological damage such as inflammation, t-Tau, and p-Tau will be released in CSF. Lower concentrations can also be found in peripheral blood. Abbreviations: CSF: cerebrospinal fluid. Created with Biorender.com.

Tau, a protein belonging to the family of microtubule associated proteins (MAPs), is involved in cellular structure and localized primarily on a neuron’s axonal tracts and, at lower levels, in glia, either oligodendrocytes or astrocytes (Figure 1a) [2]. We can account for several physiological functions for the Tau protein, mainly including microtubular assembly and axonal stabilization; Tau may also support synaptic plasticity [3]. However, the exact mechanism of microtubule stabilization and assembly remains demanding to evaluate [4]. The alternative splicing of the microtubule associated protein Tau (MAPT) gene is responsible for six different isoforms of the Tau protein [4]. Tau function also depend on phosphorylation, which decreases Tau affinity in the microtubules, ensuring a balance between assembly and disassembly in healthy neurons. However, hyper-phosphorylation may occur in neurodegenerative disease, leading to an evident neuronal loss (Figure 1b) [4]. Furthermore, upon neuronal disruption from any physiological or pathological injury, t-Tau and p-Tau can be released in the extracellular milieu and CSF (Figure 1c). Therefore, Tau can be detected in the CSF of healthy subjects as a reflection of physiological aging with different values depending on the individual’s age, but, more importantly, as a marker of central nervous system (CNS) pathology in patients with neurodegenerative diseases [5,6], representing a biomarker of axonal loss in several neurological conditions. Tau probably experiences a spontaneous clearance from the CSF to serum [7]. Thus, in the same individual, its concentrations will be higher in CSF than in serum or plasma [8] (Figure 1c). 

Tau concentration is obtained mainly with commercially available immunoassays, such as enzyme linked immunosorbent assay (ELISA), electrochemiluminescence (ECL) [9], or Western blot [10]. Recently, a novel technology, high sensitive single molecule assay (SIMOA), has been introduced [11,12]. Given its higher sensitivity than conventional ELISA, SIMOA can measure CSF proteins outside CNS [12]. 

## 3. Multiple Sclerosis

MS is a chronic disease of the CNS. The disease pathology is heavily based on inflammation and demyelination. However, in the last decades, axonal and neuronal loss have been recognized from early disease stages [13,14,15], and an increasing number of studies have focused on investigating neurodegeneration and axonal damage, which appear only partially due to inflammatory processes. Therefore, MS can be considered an inflammatory neurodegenerative disease characterized by inflammation bursts resulting in acute axonal damage and a progressive chronic neuronal loss that increases over the years. MS is highly heterogeneous, with clinical manifestations ranging from sensory or motor dysfunction to fatigue and cognitive impairment [16,17]. The core MS phenotypes are relapsing and progressive diseases, further categorized into relapsing–remitting MS (RRMS), secondary progressive MS (SPMS), and primary progressive MS (PPMS) by the rating of disease activity (clinical relapses or magnetic resonance imaging worsening) and disease progression (increased neurologic disability) [18]. Consequently, the introduction of reliable diagnostic, prognostic, and treatment response biomarkers would be essential in clinical practice. To date, diagnostic biomarkers solely rely on identifying intrathecal IgG synthesis [19,20], whereas several CSF and serum molecules have been investigated as prognostic and treatment response biomarkers [21,22], but none have yet been translated in clinical practice. Over recent years, many studies have examined total Tau (t-Tau) and p-Tau in the MS population, particularly exploring their concentration in CSF and association with clinical and radiological parameters, and only a few investigated other biological fluids. As we will analyze in detail in the following subsections, results were not always concordant, possibly based on differences in the patient populations and methodologies, which could have affected the results.

### 3.1. CSF Tau in MS: Role in the Diagnosis

Studies comparing MS patients and healthy controls (HC) often exposed discrepant results. CSF t-Tau was found increased in most studies [23,24,25], whereas few reported normal or decreased levels in small sample sizes [26,27,28,29,30]. Nonetheless, a recent meta-analysis of 17 studies confirmed that t-Tau increased in MS patients [31]. To note, several of those studies included MS and control groups without age and sex-matching [31]. Most studies were focused on evaluating t-Tau alone or combined with other biomarkers, such as Aβ, neurofilaments light chain (NfL), S100, and GFAP, thus comparing differences in MS population between neuronal and astrocytic proteins [23,25,28,32,33]. Some groups also included the analysis of p-Tau and t-Tau [7,32,33], and some detected an increased immunoreactivity of phosphorylated epitopes in progressive MS patients compared to HC. They suggested that, in the CSF of progressive MS, detection of increased p-Tau in the absence of increased t-Tau may be a highly sensitive marker of axonal damage, helpful in differential diagnosis with relapsing MS [32]. In contrast, Jaworsky et al. found similar p-Tau concentrations in MS and HC, while increased t-Tau were detected in MS patients [7]. To our knowledge, in MS, no studies have evaluated the p-Tau/t-Tau ratio. Given the possible impact of age, sex, and relapses [33], it would be crucial that comparison groups are matched for potential confounding factors in future studies. In addition, most studies were conducted with European patients, such as Pietroboni et al. [28], and these analyses must be extended to different populations [31].

### 3.2. CSF Tau in MS: A Marker of Phenotypic Variability?

Considering the extreme clinical variability of MS phenotypes, a helpful biomarker might differentiate particularly progressive and inflammatory phenotypes, which have different prognosis and treatment options. Highly heterogeneous results were obtained when categorizing patients based on phenotypes. The group of Kapaci detected high CSF t-Tau levels in progressive MS and ascribed these results to higher neurodegeneration in these forms than in relapsing MS [24]. By contrast, Jaworsky et al. observed lower CSF t-Tau levels in 14 SP patients than in 34 RR patients [7], while Terzi et al. found similar values [34]. The Jaworsky group hypothesized that SP subjects experience a decrease in neuronal volume and axonal quantity, resulting in the loss of Tau resources [7]. The limitation in stratifying people with MS is the small numbers of progressive phenotypes, especially PP, typically included in observational studies. Moreover, some studies may have considered RRMS during relapses and some during remission, possibly affecting the comparison with progressive MS [31]. 

Among relapsing phenotypes, no significant differences were observed between clinically isolated syndrome (CIS) and RR [31], but we need to consider the evolution of MS diagnostic criteria, with some CIS now being classified as RRMS [35]. Another interesting point would be to assess differences in the demographic features of progressive MS, such as age, since a linear age associated increase in CSF t-Tau has been observed in both HC and AD patients [6]. 

### 3.3. CSF Tau in MS: Role in the Prognosis

Regarding biomarkers of disease activity, NfL has been consistently associated with the presence and the number of gadolinium-enhancing lesions [31], whereas both Mori et al. and Virgilio et al. did not observe differences for t-Tau [36,37]. A correlation between magnetic resonance imaging (MRI) lesion load and t-Tau concentrations has been reported by several studies [28,38,39]. Conversely, others observed differences in t-Tau and p-Tau levels in patients with relapses, compared with stable patients [33]. An exciting possibility is that CSF Tau may be a prognostic factor for disability accumulation over time. In particular, based on their results, Frederiksen et al. proposed CSF t-Tau as a predictor of progression from CIS to defined MS [40]. Evidence pointed out that CSF Tau at diagnosis marks chronic neurodegeneration, clinical disability, and poor prognosis [2,7,34,40,41]. Most authors used the expanded disability status scale (EDSS) or, rarely, EDSS plus multiple sclerosis severity score (MSSS) as measures of disability, whereas Virgilio et al. also used age related MSSS (ARMSS) in a cohort of 100 Italian MS patients [41]. However, Edwards et al. performed three or four repeated lumbar punctures over 28 weeks in 16 SPMS patients under dimethyl fumarate (a DMT available worldwide for CIS and RRMS, but also approved for active SPMS in the US) to characterize the pharmacokinetics and CSF penetration of monomethyl fumarate—the drug active metabolite—and evaluate axonal damage biomarkers using the SIMOA assay at Quanterix (Lexington, MA) [42]. CSF t-Tau levels remained stable over the treatment, and the authors found no correlation with EDSS or MRI activity, unlike NfLs [42]. Even though published data are pretty heterogeneous in terms of disease characteristics and treatments, CSF t-Tau at diagnosis seems to correlate with disease duration and disability scores, while results on disease activity seem to be less coherent. We might speculate that t-Tau may reflect a chronic persistent axonal loss, rather than express an axonal damage caused by acute inflammation.

### 3.4. Tau and Cognitive Impairment

Cognitive impairment (CI) is frequently seen as a disabling symptom in MS patients [43], even in early disease stages [44]. Although its exact physiopathology is unknown, axonal loss from early disease stages may be partially responsible for its development [16]. Characterization of CI and the study of brain atrophy in MS in the last decades represent hot topics. Still, no specific soluble biomarkers are available for CI in MS, unlike other neurodegenerative diseases. Few studies focused on axonal damage and CI biomarkers in MS, mainly NfL, with conflicting results [16,45,46]; only one study described a correlation between CSF Aβ levels and CI [37]. In contrast, recently, Virgilio et al. observed, in 62 patients, a correlation between CSF t-Tau and information processing speed and global cognition, whereas NfL and Aβ could not discriminate CI patients [36]. Moreover, baseline t-Tau and NfL were predictors of brain atrophy after three years of follow up [47]. These preliminary results need confirmation in future studies, opening new possible uses of CSF t-Tau to evaluate CI in MS patients [48].

### 3.5. The Role of Tau Imaging in MS

Unlike other neurodegenerative diseases, evaluation of in-vivo Tau brain pathology with Tau radioligands such as [18F]AV-1451 has been poorly explored in MS. Only Zeidan et al. [48] included 12 patients with MS and 60 matched HC (for age, sex, and APOE ε4 status). Cognition was checked with four neuropsychological tests, and, as expected, MS patients displayed statistically significant differences in executive functions and language. No significant differences were observed; however, a trend for higher regional cortical AV-1451 standard uptake value ratios (SUVrs) was observed in MS patients compared to HC, and patients with longer disease duration displayed grater AV-1451 SUVrs. 

### 3.6. Peripheral Tau in MS: New Evidence

Few data on different biological fluids other than CSF in the MS population are available. CSF is less accessible and repeatable than blood, saliva, or tears. However, compared with CSF, most kits used in blood or saliva have not yet been standardized and validated for clinical uses [49]. In 2011, Bartosik et al. evaluated serum t-Tau over 24 months from mitoxantrone administration in 54 MS patients [23]. They observed a decrease in biomarker concentrations, indicating that depletive anti-inflammatory, immunosuppressive DMTs also reduce axonal loss in RR and SP patients. However, both Bartosik and Jaworsky et al. demonstrated that several patients displayed serum levels of t-Tau under the detection limit using a sandwich ELISA, even though MS patients showed high mean t-Tau serum concentrations compared to HC (Innotest hTAU-AG, Innogenetics, Ghent, Belgium) [7,23]. Therefore, as for other molecules (i.e., NfL), it is advisable to use a highly sensitive array to evaluate serum Tau concentration over time after treatment with DMTs. By contrast, Mirzaii-Dizgah et al., using another ELISA (BioAssay Technology Laboratory, Shanghai, China), detected lower Tau protein levels in the serum, but not in the saliva, of 30 MS patients compared to HC. Patients also displayed a negative correlation with EDSS [8]. Finally, Islas-Hernandez et al. used Western blotting (DCTM Protein Assay Kit, BioRad) to comply with Bartosik-Psujek and described that SP patients tend to display reduced levels of t-Tau in serum [10], in line with the results reported years before by Jaworsky et al. [7].

## 4. Amyotrophic Lateral Sclerosis

ALS is a chronic, rapidly progressive neurodegenerative disease characterized by a motoneurons’ degeneration in selected areas, such as the motor cortex, brainstem, and spinal cord, with a disease duration variable from 3 to 5 years, usually related to the worsening of muscular weakness and respiratory failure [50]. Most of the cases (roughly 85–90%) are sporadic (sALS), while a positive family history for ALS is reported in a minority of patients (fALS) (10–15%) [51]. The clinical phenotype of the disease is heterogeneous at onset and progression [52], and half of the patients display CI, ranging from mild CI to FTD [53,54]. 

Most ALS patients (>95%) have transactive DNA binding protein of ~43 kDa (TDP-43) inclusions in postmortem studies, whereas Tau is not noteworthy [55] (except for Guam ALS/parkinsonism, which is mainly a tauopathy [56]). No specific diagnostic tests are available for ALS, and the diagnosis is primarily reached by excluding secondary/acquired forms [57]. Indeed, there are no precise prognostic markers, including fluid biomarkers, available for ALS, and, frequently, all of the proposed biological markers give confounding and conflicting results. Only NfLs have been recently proven to mark neurodegeneration and clinical progression in ALS, with the highest levels in ALS rather than FTD [58,59].

### 4.1. CSF Tau in ALS: Role in the Diagnosis

In recent years, several cytoskeletal proteins have emerged as candidate ALS biomarkers. Among them, the role of the Tau protein in motoneuron diseases has been investigated in a few studies, without achieving univocal results.

Overall, some studies showed a significant difference in CSF t-Tau and p-Tau levels between ALS patients and HC, both with a diagnostic and prognostic role, while other works fail to demonstrate a correlation between the protein and the disease. In a small preliminary study, high Tau levels were shown in 70% of patients compared to controls, with the highest Tau levels in the early disease stage [60]. Similar results were confirmed by the same group years later, showing increased levels of the Tau protein both in comparison with HC and other MND forms [61]. After analyzing p-Tau and t-Tau in CSF separately, p-Tau was not significantly reduced in ALS patients compared with HC. However, t-Tau was increased considerably, with a consequent reduction of the p-Tau/t-Tau ratio [62]. Integrating the ratio with the TDP-43 level in a combined formula, this score resulted in a specific and sensible index for diagnosis [63]. In line with these findings, a recent Italian study observed that ALS patients showed significantly higher CSF t-Tau and a lower p-Tau/t-Tau ratio than controls (*p*-value < 0.001). However, no differences in p-Tau levels were detected [64]. In another cohort, ALS patients had higher levels of t-Tau and lower p-Tau/t-Tau ratio than ALS mimics and other not neurodegenerative diseases, although without ligh levels of sensibility and specificity [65,66].

### 4.2. CSF Tau in ALS: Role in the Prognosis

ALS progression and survival are highly variable among patients, without clear markers able to predict it. Can Tau help in this regard? The Grossman group published a consistent paper in 2010, showing deficient CSF levels of p-Tau in ALS and revealing that the p-Tau/t-Tau ratio could distinguish individuals with ALS from HC and individuals with tauopathies. Additionally, in the same study, low p-Tau levels and p-Tau/t-Tau ratio correlated with clinical measures of disease such as the ALS functional rating scale-revised (ALSFRS-R) score and mini-mental state examination (MMSE), and with MRI measures of reduced white matter fractional anisotropy in the corticospinal tract and prefrontal cortex in ALS subgroups [67]. In terms of correlation with upper motor neuron involvement, these results were further confirmed with a study that showed that a low p-Tau/t-Tau ratio was associated with global grey matter brain atrophy and diffuse white matter integrity loss, highlighting that this index can be a marker of central motor degeneration [68]. A recent Italian study [64] showed, by multivariate analysis, that t-Tau acts as an independent negative predictor of overall survival, and high levels of this biomarker are associated with a fast rate of ALSFRS-R score progression. In another above cited cohort, the authors demonstrated that CSF t-Tau correlated with progression rate and muscle strength indexes (e.g., the sniff nasal inspiratory pressure). On the contrary, CSF p-Tau was not related to any ALS clinical feature [65]. In addition, the group of Blasco correlated the Tau level with functional score and disease progression, with possible prognostic meaning, and multivariate analysis revealed that ALSFRS-R at baseline was associated with the p-Tau/t-Tau ratio. Both measurements independently correlated negatively with ALSFRS-R variation over the disease course; consequently, the p-Tau/t-Tau ratio correlated positively with ALSFRS-R changes [69]. Finally, the role of t-Tau as a marker of rapid disease progression was recently confirmed, especially integrating this marker with NfL [70].

These studies, albeit heterogenous in terms of results, methods, and sample size, indicate that p-Tau has poor sensitivity and specificity, but t-Tau, and especially the p-Tau/t-Tau ratio, have moderate sensitivity and specificity for both the diagnosis and prognosis of motoneuron diseases. Tau levels may help to confirm ALS diagnosis and establish the prognosis despite the lack of high specificity, especially when integrated with other fluid biomarkers. However, further studies are mandatory to explore the pathophysiological and neuropathological mechanisms associated with these findings and confirm the clinical value.

## 5. Frontotemporal Spectrum Disorder

FTSD is an insidious neurodegenerative syndrome resulting from progressive language, behavioral and executive deficits. The disorder is the most frequent early onset dementia and, globally, the third most common form of dementia across all age groups after AD and Lewy bodies dementia (DLB) [71]. Clinically, FTSD is classified into three clinical variants, one behavioral and two with language alterations. The first one is associated with early behavioral and executive deficits (bvFTD); the other two include the nonfluent variant (PNFA), with progressive deficits in speech and grammar, and semantic dementia (SD), which is characterized by a progressive deficit of semantic knowledge and naming. Progressively over the disease course, the symptoms of the three variants can converge and overlap as the damage spreads in the frontal and temporal lobes, showing severe cognitive impairment, parkinsonism, and motoneuron disorders. The disease duration varies from 8 to 10 years, and death is mainly due to cachexia and infections [72]. FTSD patients have multiple neuropathological phenotypes, including the microtubule associated protein tau (MAPT), the TDP-43, or the fused in sarcoma (FUS) protein. In frontotemporal lobar degeneration (FTLD), the commonest subtypes of Tau related disease are Pick’s disease (30%), corticobasal syndrome (CBS) (40%), and progressive supranuclear palsy (PSP) (30%) [73]. For FTSD, an extended clinical assessment associated with language, socio-emotional functioning, cognition, and neuroimaging evaluation (brain magnetic resonance to evaluate the atrophy specific patterns and positron emission tomography for the brain metabolism) can support the diagnosis [74].

### 5.1. CSF Tau in FTLD: Is a Pathological Role Possible?

Pathologically, it has been shown that FTSD has inclusions mainly containing either Tau or TDP-43. However, a clinicopathological correlation is not easy, and diagnosing the correct proteinopathy during life (without autoptic data) is hard. To date, this is possible only when a genetic cause is present (e.g., mutations in MAPT and Tau pathology, in GRN and C9orf72 and TDP-43 pathology) or for specific phenotypes (e.g., the ALS-FTSD spectrum is primarily associated with TDP-43 pathology).

Overall, the CSF p-Tau/t-Tau ratio has high accuracy in discriminating FTLD patients compared to HC and AD, and it is driven by elevated t-Tau levels in patients [75]. However, it seems that FTSD CSF Tau levels are not different in FTLD patients with underlying Tau pathology (e.g., with MAPT mutations) compared with Tau negative or sporadic FTLD. The first published study in this regard, in 2003, showed only a mild increase in t-Tau levels in FTSD patients compared with nondemented controls and lower levels in the subgroup with Tau mutations compared with AD patients. Moreover, p-Tau was not significantly different in FTSD patients based on genetic status, compared with HC [76]. Similarly, Rohrer and colleagues demonstrated that CSF t-Tau concentrations were considerably higher in the FTLD-TDP-43 and FTLD-Tau groups than in controls, without differences between the patient groups. Similar results were obtained for the p-Tau/t-Tau ratio, which was significantly lower in the FTLD-TDP-43 and FTLD-Tau groups than in controls, but without any difference between the two patient groups [77]. Despite this, the capacity of the ratio to distinguish the pathological phenotypes is debated, and other groups found a significantly reduced CSF p-Tau/t-Tau ratio in FTLD-TDP-43 compared to FTLD-Tau, with a decent predictive value [78,79,80]. The decreased ratio in FTLD-TDP-43 seems to be driven by low levels of p-Tau in FTLD-Tau, while t-Tau levels were similar [75], but a possible copresence of MND in the FTLD-TDP-43 forms must be considered.

### 5.2. CSF Tau in FTLD: A Marker of Phenotypic Variability?

Considering the extreme clinical variability of FTSD phenotypes, a helpful biomarker would allow to differentiate particularly bvFTD and PNFA and SD, which can have different prognosis and experimental and symptomatic treatment options. For example, regarding differences between phenotypes, results are highly heterogeneous. The group of Bittner described higher t-Tau levels in SD than HC and CBS/PSP patients. Additionally, p-Tau was higher in all FTSD than CBS/PSP subjects but still within the normal range. Furthermore, unremarkable results were detected by comparing p-Tau and t-Tau in PNFA and non-PNFA patients [81]. By contrast, Meeter et al. observed that the p-Tau/t-Tau ratio can discriminate FTSD from controls, but not the clinical subtypes, except for cases with concomitant MND involvement [75]. Although not easy because of the rarity of neurodegenerative language disorders, this investigation certainly deserves to be developed, especially to distinguish the different forms of aphasia and the AD phenotype.

### 5.3. Tau in FTLD: Role in the Prognosis

Few data are accessible on the prognostic role of CSF Tau in FTLD patients. In 2014, an Italian group demonstrated that FTSD patients with high CSF Tau levels (≥400 pg/mL) had shorter survival than those with low CSF Tau levels [82]. In 2018, Ljubenkov et al. showed that high baseline levels of CSF t-Tau and p-Tau predicted a fast rate of worsening only in bvFTD patients. Additionally, p-Tau had a similar predictive value as NfL in bvFTD, while t-Tau had predictive value only in PNFA subjects, likely correlating specific differences in Tau production, post-translational changes, or degradation due to the different FTLD subtype [83]. By contrast, moving to plasma, it was demonstrated that p-Tau levels were not correlated with neuropsychological, behavioral, and functional measures and did not help to monitor disease severity or predict prognosis throughout the FTLD spectrum [84]. Similarly, the group of Rojas demonstrated that high baseline plasma t-Tau concentrations were associated with a fast decline in the bvFTD and PSP subgroups of FTLD, but introducing the plasma t-Tau in a Cox model for survival did not change the event probability [85]. Since the p-Tau/t-Tau ratio is a nonspecific marker of neuronal loss, it is not surprising that it has a limited role in subtyping different FTSD phenotypes, across bvFTD, PNFA, and SD in several series. Nevertheless, despite other markers, many reports indicate that the p-Tau/t-Tau ratio is relatively specific in differentiating TDP-43 from Tau pathology, helping the choice of disease-modifying drugs that can target the specific underlying pathological mechanism. At this point, it is clear that further studies may improve understanding of the essential pathophysiological role of plasma Tau levels, but, to date, the extensive overlap with those of HC limits the diagnostic utility.

### 5.4. Peripheral Tau in FTLD: New Evidence

Current CSF biomarkers, including dosage of TDP-43 and Tau, cannot accurately identify the underlying phenotype and proteinopathy in vivo in FTSD, and, therefore, novel measures should be identified. Hence, the need to investigate plasma biomarkers, including the Tau assay. Plasma Tau has been measured in several studies, observing an increment in plasma Tau levels in patients with CI, but independently from AD or FTSD. In 2018, Rohrer measured plasma Tau concentrations in a large group of FTSD patients with an ultrasensitive detection method, and showed that bvFTD and PNFA displayed higher plasma Tau concentrations than controls. In addition, upon stratifying for genetic data, only the MAPT group had significantly increased concentrations of plasma Tau. On the contrary, there were no correlations of Tau levels with brain volumes, serum NfL concentrations, or disease duration [86]. Another work, including patients with different FTSD variants, showed increased plasma Tau levels in all clinical FTSD subgroups, but—in terms of genotype—only in MAPT mutations [87].

### 5.5. The Role of Tau Imaging in FLTD

Regarding Tau-PET with [18F]AV-1451, recent clinical trials aspired to diminish pathological protein aggregates in neurodegenerative diseases. Similar to the role of Aβ PET in clinical trials for AD, an imaging marker able to quantify Tau can help develop anti-Tau drugs, supporting participant selection, early intervention, and assessment of proper goals.

To date, in vitro studies with the AV-1451 tracer in non-AD patients obtained contrasting results [88,89]. However, the number of in vivo studies has been growing recently: one of the most consistent studies, published in 2019 by the group of Rabinovici, described the [18F]AV-1451 PET findings of FTSD patients with different clinical and genetic features. A single subject analysis reported a low level pattern of [18F]AV-1451 binding able to coordinate Tau pathology’s expected anatomical distribution and frequency. For example, nfvPPA patients showed increased binding in the left inferior frontal gyrus, CBS patients in frontal white matter, and 50% of bvFTD in frontotemporal areas. However, compared with controls, they frequently did not observe regions with significant retention or a modest overlap between patients and HC [90]. Concerning MAPT mutation, the same group observed an increased [18F]AV-1451 retention in bilateral temporal lobes [90], confirming the results of previous studies [91,92]. In addition, a new promising tracer, the 18F-MK-6240, was recently used in an elegant work to bind Tau in vivo in genetic FTSD. This group reported a mild but significant binding of tracer in amyloid negative MAPT mutation patients, highlighting that a positive [18F]MK-6240 tau-PET does not imply with certainly an AD diagnosis [93], and pointing towards a potential use of Tau tracers as a biomarker in tauopathies beyond AD, even with some limitations due to the modest affinity.

## 6. Parkinsonian Syndromes

Parkinsonian syndromes, particularly Parkinson’s disease (PD), are prevalent neurodegenerative disorders. Clinically, PD presents bradykinesia, resting tremor, rigidity, postural instability, and nonmotor symptoms [94]. The pathological hallmark of the disease is the progressive depauperation of dopaminergic neurons in the substantia nigra pars compacta (SNc) and the formation of Lewy bodies (LBs) in the residual neurons. LBs consist of aggregated forms of the α-synuclein protein (α-syn), which contribute to the pathogenesis of PD [95]. Even though exact mechanisms underlying the α-syn aggregation are not fully understood, both active and passive immunization strategies targeting this protein have been developed. Unfortunately, immunization with α-syn was effective in animal PD models [96,97], but phase 2 clinical trials failed to meet primary endpoints. These findings questioned the role of α-syn aggregates in cell death and suggested that protein aggregation may be the consequence of damage, rather than being the primary cause of neurodegeneration, and stimulated researchers to find other contributors to PD pathology. In this context, an underappreciated component is the Tau protein, which shares some properties with α-syn, as they are both brain proteins with prion like characteristics. Furthermore, genome wide association studies indicated that single-nucleotide polymorphisms (SNPs) in MAPT and SNCA genes are common risk factors for PD [98,99].

### 6.1. The Role of the Tau Protein: Evidence from Animal Models and Neuropathology

In neurodegenerative disorders, interactions between α-syn and Tau can promote protein fibrillization and stimulate the creation of pathological inclusions [100]. Evidence confirmed that α-syn contributes to Tau phosphorylation, mainly via the glycogen synthase kinase 3beta (GSK-3β) in the PD animal model [101] and α-synuclein-overexpressing transgenic mice overexpressed α-syn, p-Tau, and p-GSK-3β. Moreover, these proteins are co-localized in large inclusion bodies, similar to LBs [102]. Those results were also replicated in human models: Arima et al. found the co-localization of Tau and α-syn in PD brains [103], while Compta et al. reported a combination of Lewy and AD type inclusions as dementia’s pathological correlates [104], corroborating Tau’s involvement in PD. Furthermore, Tau pathology was explored in PD patients receiving fetal neural allografts as cell replacement therapy: Cisbani et al. found that hyperphosphorylated Tau can be detected in grafted tissue 16 years post-transplantation [105]. Similarly, Ornelas et al. showed neuronal perikaryal inclusions of phosphorylated α-syn and Tau in the graft tissue [106], suggesting that both α-syn and Tau pathology can spread from the host to the graft.

### 6.2. Tau Protein in PD: Role in the Diagnosis and Prognosis

Published literature has widely investigated CSF Tau levels as biomarkers for PD diagnosis and progression. Higher CSF t-Tau levels were reported in nondemented PD patients compared with HC [99,107] and mainly in the subgroup of patients with short disease duration, implying that the initial PD stages are crucial for neurodegenerative changes. However, 20 patients (62.5%) were treated with levodopa in the study, which opens the question of possible treatment interference. In this regard, it was proved that CSF α-syn levels at 12 months were lower in PD patients treated with dopamine replacement therapy, especially dopamine agonists, but no significant relationships were found with t-Tau and p-Tau levels [108]. A total of 109 newly diagnosed, drug naive, and cognitively spared PD patients displayed significantly reduced CSF Aβ but not t-Tau or p-Tau, compared to controls [109]. Another Italian study found that PD patients showed similar CSF t-Tau and p-Tau as controls, but lower levels compared to dementia with Lewy bodies (DLB), AD, and FTSD subjects [110]. The same research group subsequently found that greater diagnostic accuracy in detecting PD patients could be achieved by combining oligo/total α-syn and Aβ/tau ratios, further confirming the role of Tau in disease assessment [111]. How these biomarkers varied across different disease time points was evaluated by Mollenhauer et al. by sampling CSF α-syn, t-Tau, p-Tau, and Aβ42 levels at baseline and after 6 and 12 months in a large cohort of PD patients and matched HC. Results showed that t-Tau remained stable, and there was a slight increase in p-Tau in PD patients, but there was no correlation with motor scores or dopamine imaging [108]. Conversely, another study showed that p-Tau levels were lower in 112 nondemented PD patients than in HC at baseline and increased significantly after one year, whereas t-Tau levels did not show significant longitudinal changes after the same follow up time [112].

### 6.3. Tau Protein in PD: A Marker of Phenotypic Variability?

#### 6.3.1. Tau Protein and Motor Symptoms in PD

The relationship between Tau levels and PD motor and nonmotor characteristics has been assessed by several studies. CSF Tau levels helped to detect tremor dominant (TD) PD since non-TD patients displayed higher levels of t-Tau and an increased Tau/Aβ42 index [113,114]. In the context of the Parkinson’s progression marker initiative (PPMI), others found decreased CSF t-Tau levels and a correlation with increased motor severity. Furthermore, low CSF Aβ42 and p-Tau levels were associated with the postural instability gait disturbance (PIGD) dominant phenotype [115]. A subsequent PPMI study involving 660 PD patients partially confirmed these findings [116]. Intriguingly, genetic background might also be relevant, since Vilas et al. [117] described that, in the subgroup of patients with a mutation in the leucine rich repeat kinase 2 (LRRK2) gene, CSF t-Tau levels were higher in PIGD-PD than TD-PD, whereas no differences were detected when all patients (with or without LRRK2 mutation) were analyzed together. Another study aimed to identify distinct subgroups via cluster analysis and found that patients with diffuse malignant PD had the lowest level of Aβ and Aβ/t-Tau ratio in CSF. However, similar t-Tau and p-Tau levels were detected in the mild motor predominant and intermediate phenotypes [118]. In a longitudinal analysis of 403 PD patients, the DATATOP Investigation Group reported that the rate of change in CSF t-Tau levels significantly correlated with the rate of motor unified Parkinson’s disease rating scale (UPDRS) change. Similar findings were observed between CSF t-tau/Aβ42 variations and modifications in total and motor UPDRS [119]. In summary, distinct PD phenotypes and the severity of motor symptoms may underlie specific biomarker dynamics, but the role of Tau has not been unequivocally established.

#### 6.3.2. Tau Protein and Nonmotor Symptoms in PD

Regarding nonmotor symptoms, an increased CSF t-Tau/Aβ42 ratio was described in PD patients with REM sleep behavior disorder [120]. The role of Tau has been primarily explored in the context of CI and PD dementia (PDD) [121]. Several studies showed increased CSF levels of t-Tau and p-Tau in PDD subjects compared with HC [122,123]: PD patients with a high p-Tau and p-Tau/Aβ42 ratio developed subsequent decline in cognitive tasks, particularly memory and executive functions [124]. Similarly, CSF t-Tau/Aβ ratio was associated with Montreal cognitive assessment (MoCA) score at two years in 390 PD patients [125], and t-Tau/Aβ42, t-Tau/α-syn, t-Tau/Aβ42+α-syn, and Aβ42/t-Tau ratios showed a significant association with the risk of progression to dementia over a 3-year follow up [126]. Nonetheless, heterogeneous results have been described, since another study reported that CSF p-Tau concentrations were 20% lower in cognitively normal-PD and CI-PD without dementia than in age matched HC, but levels of t-Tau were not changed in PDD patients [127]. Similarly, Bibl et al. did not find significant differences in t-Tau CSF levels between PDD and controls [128], and there were no correlations between Tau levels and cognitive measures [109]. A longitudinal study [129] evaluating CSF biomarkers in 415 PD patients with ten years of follow up failed to find any significant association between t-Tau, p-tau, and MoCA scores. These findings might support the hypothesis that PD cognitive dysfunction is associated with an AD like CSF biomarker profile [130,131], but caution should be taken when considering CSF Tau measurements in PD. 

### 6.4. Tau Protein in Atypical Parkinsonian Syndromes: Role in Diagnosis

Tau protein evaluation might also be relevant in the differential diagnosis between PD and atypical parkinsonism disorders (APD). APDs are a group of heterogeneous neurodegenerative diseases such as multiple system atrophy (MSA), progressive supranuclear palsy (PSP), corticobasal syndrome (CBS), and DLB. Among α-synucleinopathies, MSA is a progressive neurodegenerative disease characterized by autonomic disturbances, pyramidal, parkinsonian or cerebellar features [132]. Regarding DLB, the diagnosis is suggested by the appearance of parkinsonian symptoms, dementia, hallucinations, and delusions, frequently fluctuating during the day. Clinical diagnostic criteria have been delineated for both DLB and PDD, and the two diseases are differentiated based on the 1-year rule of cognitive and motor symptoms’ occurrence [133]. PSP is a tauopathy characterized by atrophy of the dorsal midbrain and a rapidly evolving parkinsonism with unsteadiness of gait, falls, and alteration of vertical eye movements, even though multiple clinical phenotypes have been included in the diagnosis [134]. CBS consists of various asymmetrical parkinsonian features, dystonia, myoclonus, and different underlying pathological substrates such as corticobasal degeneration (CBD), AD, PSP, and FTSD-TDP-43. Over the years, the terms CBS and CBD have been used interchangeably, but the latter refers to the specific pathological entity of a 4-repeat tauopathy [135]. Due to possible clinical overlap in the initial stages of the disease, the distinction between PD and other parkinsonian syndromes can be challenging, thus prompting researchers to identify reliable biomarkers.

Hansson et al. [136] found increased CSF t-Tau levels in MSA and CBS patients compared with PD, and lower t-Tau levels in PSP than MSA and HC, whereas CSF p-Tau levels were significantly lower in MSA and PSP than in controls, but no differences were detected compared with PD patients. In the validation cohort of the same study [136], however, there were no differences between groups in t-Tau levels, and significantly lower levels of p-Tau were observed just in PSP patients compared with HC and PD. Furthermore, in the first cohort, high levels of NfL were associated with high levels of CSF t-Tau, but this was not confirmed in the validation cohort. The discrepancies of these results have challenged these biomarkers’ diagnostic utility, as highlighted in other studies. Sussmuth et al. observed higher CSF t-Tau in PSP-parkinsonism (PSP-P), MSA-parkinsonism (MSA-P), and MSA-cerebellar (MSA-C) when compared with PD. Patients with PSP-Richardson’s syndrome (PSP-RS) had normal t-Tau levels. By contrast, PSP-P patients displayed significantly higher levels than PSP-RS, PD, and HC. CSF p-Tau was not informative in differentiating APD. However, P-tau/T-tau ratios were lower in PSP and MSA when compared with PD [137]. Similarly, several studies confirmed higher CSF t-Tau in MSA than PD [138,139,140,141]. In contrast, CSF t-Tau and p-Tau did not help to discriminate between MSA and PD in other cohorts [142,143]. Nonetheless, the good clinical accuracy of Tau/α-syn ratio in discriminating DLB patients was reported [143], in line with the increase in t-Tau and p-Tau found in DLB [144,145]. Evidence suggested that CSF Tau levels are higher in DLB than in PSP and CBD [145] and compared with other synucleinopathies [123,143], but significantly lower than in AD [146]. Furthermore, recent studies reported substantially lower plasmatic p-Tau levels in DLB than in AD, and an association with the progression of cognitive decline [58]. Even though Tau level could be affected by age [6,104,142], it might still be helpful as a prognostic factor.

### 6.5. The Role of Tau Imaging in Parkinsonian Syndromes

The contribution of brain Tau aggregates can be evaluated in vivo using specific radioligands, such as [18F]AV-1451, also known as [18F]T807 [147,148]. This radiotracer has been extensively used in parkinsonian disorders: a reduction in PD patients of its volume of distribution compared with controls reflected the loss of pigmented neurons in the SN [149]. The relationship between Tau imaging and CI has also been widely investigated, and Tau aggregates in PD correlate with the severity of CI in both DLB and PDD [150,151], with a more significant Tau burden in DLB than PDD tissue [152]. Nonetheless, Tau binding of [18F]AV-1451 was lower than binding predicted from pathological studies: for instance, Winer et al. reported no differences in the patterns of Tau deposition in 15 cognitively normal PD, 14 cognitively impaired PD, and 49 cognitively normal HC [153]. Similarly, it was suggested that Tau pathology, evaluated through [18F]AV-1451, is uncommon in PD with mild CI and no significant correlation with cognitive dysfunction [154], at baseline as well as after a 2-year follow up [155], was observed. However, cortical Tau aggregates were found in DLB and CI PD, suggesting that patients with DLB display a spectrum of Tau pathology [156]. Regarding other APDs, several studies observed in PSP-RS elevated radioligand uptake mostly in subcortical structures, including midbrain, dentate nucleus, thalamus, subthalamic nucleus, globus pallidus, and striatum, compared to controls [157,158]. A multicenter study involving 33 PSP, 26 PD, and 46 HC patients found that [18F]AV-1451 uptake in the globus pallidus had the highest accuracy in PSP differential diagnosis [159]. Moreover, different degrees and patterns of Tau uptake may reflect the presence or absence of AD pathology in CBS patients [160]. In summary, Tau imaging may be valuable in parkinsonian disorders, even though some limitations due to potential off target binding should be considered when interpreting the results [161].

### 6.6. Future Perspectives: Tau Based Therapies

The involvement of Tau in several neurodegenerative diseases makes it a suitable therapeutic target in the evolving scenario of potential disease-modifying treatments. Currently developed strategies include active Tau vaccines, monoclonal antibodies, the development of microtubule-stabilizing agents, and post-translational modifications [162]. BIIB092 (Gosuranemab) lowered CSF Tau levels in PSP [134] and showed a favorable safety profile in a phase 1 trial [163], but it did not meet the primary and secondary endpoints in the phase 2 PASSPORT study [164]. Davunetide, a neuropeptide with microtubule-stabilizing properties, failed to confirm the efficacy reported in animal models [165]. Another approach using Tideglusib, an inhibitor of GSK-3β, was unable to show significant clinical differences in a multicenter, randomized, double blind, placebo controlled trial [166]. In the context of Tau based therapies in PD, the inhibitors of GSK-3β (L803-mt and AR-A014418) reduced Tau phosphorylation and spared dopaminergic neurons from cell death in mesencephalic cultures [167,168].

## 7. Final Remarks and Conclusions

**Figure 2:**
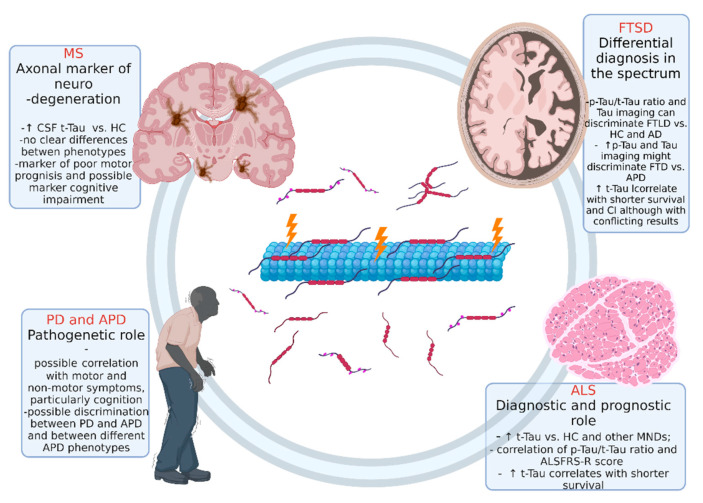
Overview of the role of Tau protein in different non-AD neurodegenerative disease. Abbreviations: ALS: amyotrophic lateral sclerosis, FTSD: frontotemporal spectrum disorder; MS: multiple sclerosis; PD: Parkinson’s disease. Figure created with Biorender.com.

We present the current principle knowledge on Tau protein in different non-AD neurodegenerative diseases. Table 1 and Figure 2 summarize the main findings for all the discussed neurodegenerative diseases.

The existing expertise ranges from conditions where Tau plays neuropathological roles (FTD and certain APD) to newly recognized neurodegenerative diseases (i.e., MS) where the Tau protein represents an interesting axonal damage biomarker that will be further investigated in the future. Several lines of evidence support the crucial role of Tau in the pathogenesis of PD and other parkinsonian disorders. Nonetheless, multiple challenges still have to be overcome to obtain reliable imaging and biofluid markers. Developing trackers of early diagnosis and disease progression will provide, indeed, invaluable help for the research of novel therapeutic strategies. ALS Tau levels may help in the diagnostic processes and define patients’ prognosis, particularly when associated with other markers. However, further studies are mandatory to explore the pathophysiological and neuropathological mechanisms related to the exposed findings. The most exciting role of Tau and ratios in FTD could be the differentiation between the subtype of the spectrum, particularly TDP-43 patients from Tau pathology, leading to the development of specific and distinct DMTs. However, several limitations to the presented literature need to be pointed out. Potential confounders of the studies include preanalytical and analytical variables, different criteria for control selection, clinical heterogeneity of patient cohorts, and the different sample size of the study groups. Nonetheless, highly standardized procedures could help reduce the variability in CSF and, more importantly, in serum Tau measurement, thus effectively defining the usefulness of this biomarker.

## Figures and Tables

**Table 1:** Overview of the principal findings regarding Tau protein in non-AD neurodegenerative diseases.

Disease	Role	Methods	Findings	References
MS	Diagnosis	CSF	-t-Tau ↑ in patients vs. HC (confirmed by a metanalysis)	[7,23,24,25,31]
			-t-Tau ↓ in patients vs. HC or no differences	[26,27,28,29,30]
			-similar p-Tau levels in MS vs. HC	[7]
		Serum	-t-Tau ↑ in patients vs. HC (some patients with undetectable values)	[23]
			-t-Tau ↓ in patients vs. HC	[8]
		Saliva	-no differences between MS vs. HC	[8]
		PET	-differences not s.s in [18F]AV-1451 in MS vs HC	[48]
	Phenotypic variability	CSF	-similar t-Tau in relapsing and progressive phenotype	[31,34]
			-↓ t-Tau in progressive phenotype	[7]
			-↑ t-Tau in progressive phenotype	[24,41]
			-No differences between relapse vs. remission	[31,33]
			-Similar t-Tau in CIS and RR MS	[31]
	Prognosis	CSF	-No association with gd+ lesion	[36,37]
			-Positive correlation with LL	[28,38,39]
			-↑ Tau predicts conversion from CIS to CDMS	[40]
			-Marker of poor prognosis, correlation with disease duration and higher EDSS, MSSS and ARMSS	[2,7,34,40,41]
		Serum	-no correlation with EDSS, MRI activity, Tau levels stable under low-efficacy DMT	[42]
			-↓ t-Tau under immunosuppressive DMT	[23]
		PET	-↑ [18F]AV-1451 in MS with longer disease duration	[48]
	Cognition	CSF	-No correlation with cognition	[37]
			-Correlation with IPS and global cognition	[36]
			-t-Tau predictor brain atrophy at 3years	[47]
ALS	Diagnosis	CSF	-↑ t-Tau vs. HC (mainly in the earlier stages);	[60,62,63]
			-↑ t-Tau in ALS vs. other MNDs;	[61,65,66]
			- no differences in p-Tau in ALS and HC	[62,64]
	Prognosis	CSF	-↓ p-Tau/t-Tau ratio correlated with ALSFRS-R score;	[67,69]
			-↓ p-Tau/t-Tau ratio correlated with WM anisotropy;	[67,68]
			-↑ of t-Tau correlates with shorter survival	[64]
			-p-Tau alone is not related to any ALS clinical feature	[65]
FTLD	Diagnosis	CSF	-high accuracy of p-Tau/t-Tau ratio in discriminating FTLD vs. HC and AD	[75]
			-similar Tau levels in FTLD Tau + vs. FTLD Tau-	[76,77]
		PET	-↑ [18F]AV-1451 and [18F]MK-6240 in MAPT mutated patients	[90,91,92,93]
	Phenotypic variability	CSF	-p-Tau/t-Tau ratio discriminates FTSD from HC, but not the clinical subtypes (except cases with MND)	[75]
			-↑ p-Tau in all FTSD vs. CBS/PSP, ↑ t-Tau levels in SD patients	[81]
		PET	-[18F]AV-1451 might discriminates different phenotypes	[90]
	Prognosis	CSF	-↑ t-Tau levels correlate with shorter survival	[82,85]
		Plasma	-↑ Tau in CI patients, independently from AD or FTSD	[81,87]
			-no correlation p-Tau with neuropsychological, behavioral, and functional measures	[84]
		Serum	-no correlations of Tau levels with brain volumes, serum NfL concentrations, or disease duration	[86]
PD	Pathology	Animal and Human model	-α-syn induces Tau phopshorilation, Tau inclusions found in PD brains	[101,102,103,104,105,106]
	Diagnosis	CSF	-t-Tau ↑ in patients vs. HC	[107]
			-no correlation t-Tau, p-Tau with dopamine imaging	[108]
			-no differences t-Tau or p-Tau vs. HC	[109,110,112]
			-lower t-Tau and p-Tau, t-tau/Aβ1–42, p-tau/Aβ1–42 ratios in PD vs HC	[116]
			-Aβ/tau ratios useful for discriminating PD	[111]
			-↓ p-Tau in non-demented PD vs. HC at T0, ↑ after 1-y	[112]
		PET	-↓ [18F]AV-1451 in SN vs. HC	[149]
	Prognosis	CSF	t-Tau ↑ in patients with short disease duration	[107]
			- no changes of t-Tau and p-Tau levels under treatment	[108]
	Phenotypic variability (motor symptoms)	CSF	-↑ t-Tau and ↑ Tau/Aβ42 index in non-TD vs. TD PD	[113,114]
			-↓ t-Tau correlates with ↑ motor severity	[115,116]
			-no correlation t-Tau, p-Tau with motor scores	[108]
			-↓ p-Tau associated with PIGD-dominant phenotype	[115,116]
			-↑ Tau in PIGD-PD vs. TD-PD in LRRK2 mutated PD	[117]
			-↓ Aβ/t-Tau ratio in diffuse malignant PD. -similar t-Tau and p-Tau in mild and intermediate motor phenotypes	[118]
			-correlation t-Tau, t-tau/Aβ42, and motor and total UPDRS change.	[119]
	Phenotypic variability (non-motor symptoms)	CSF	-↑ t-Tau/Aβ42 ratio RBD PD	[120]
			-↑ t-Tau p-Tau Tau/Aβ42 ratio in PDD vs. HC or not demented PD	[122,123,124]
			-t-Tau/Aβ42, t-Tau/α-syn, t-Tau/Aβ42+α-syn, and Aβ42/t-Tau ratios correlate with cognition	[125,126]
			-↓ p-Tau in cognitively normal-PD and CI-PD without dementia vs. HC	[127]
			-no differences in t-Tau in PDD patients vs. HC, no correlation with cognition	[109,127,128,129]
		Neuropathology	Correlation with cognition	[150,151]
		PET	No correlation with cognition	[153,154,155]
APD	Diagnosis	CSF	↑ t-Tau and p-Tau, tau/ α syn in DLB vs- HC and PD	[110,143,144,145]
		CSF and serum	↑ t-Tau in DLB vs. PSP and CBD, but lower than AD	[145,146]
		CSF	-↑ t-Tau in MSA and CBS vs. PD	[136,138,139,140,141]
			↓ t-Tau in PSP vs. MSA	[136]
			-↓ p-Tau levels in MSA and PSP vs HC, similar vs. PD	[136]
			-no differences between APD in t-Tau and p-Tau	[136,142,143]
			-normal t-Tau levels in PSP-RS. ↑ PSP-P vs. PSP-RS, PD, HC. p-Tau not informative. ↓ P-tau/T-tau ratios in PSP and MSA vs PD	[137]
		PET	-↑ [18F]AV-1451 PSP-RS vs. HC and PD.	[157,158,159]
			-[18F]AV-1451 uptake reflects the presence of AD pathology in CBS patients	[160]
	Prognosis	PET	-Tau imaging correlates with cognition in both CI-PD and DLB	[156]

Abbreviation: moto predominant phenotype; AD: Alzheimer’s disease; ALS: amyotrophic lateral sclerosis; APD: atypical parkinsonism disorders; ARMSS: age related MS severity score; Aβ: amyloid-beta; CBD: corticobasal degeneration; CBS: corticobasal syndrome; CI-PD: cognitively impaired-PD: CIS: clinically isolated syndrome; CSF: cerebrospinal fluid; DLB: dementia with Lewy Bodies; DMT: disease-modifying treatment; EDSS: expanded disability status scale; FTSD: frontotemporal spectrum disorder; FTLD: frontotemporal lobar degeneration; HC: healthy controls; LB: Lewy body; LRRK2: leucine rich repeat kinase 2; MND: motor neuron disease; MS: multiple sclerosis; MSA: multiple system atrophy; MSSS: MS severity score; NfL: neurofilaments light chain; PDD: Parkinson’s disease dementia; PIGD: postural instability gait disturbance; PNFA: progressive nonfluent aphasia; PSP: progressive sopranuclear palsy; PSP-P: progressive sopranuclear palsy-parkinsonism; PSP-RS: progressive sopranuclear palsy-Richardson’s syndrome; p-Tau: phosphorylated Tau; RR: relapsing–remitting; SD: semantic dementia; TD: tremor dominant; t-Tau: total Tau; UPDRS: unified Parkinson’s disease rating scale; α-syn: α-synuclein.

## Data Availability

Not applicable.

## Abbreviations

AD	Alzheimer’s disease
ALS	amyotrophic lateral sclerosis
APD	atypical parkinsonism disorders
ARMSS	Age related multiple sclerosis severity score
Aβ	amyloid-beta
bvFTD	behavioral variant of frontotemporal dementia
CBD	corticobasal degeneration
CIS	clinically isolated syndrome
CSF	cerebrospinal fluid
DMT	disease-modifying treatment
DLB	Lewy bodies dementia
ECL	electrochemiluminescence
EDSS	expanded disability status scale
ELISA	enzyme linked immunosorbent assay
fALS	familial ALS
FTSD	frontotemporal spectrum disorder
FTLD	frontotemporal lobar degeneration
GSK-3β	glycogen synthase kinase 3beta
HC	healthy controls
LB	Lewy body
LRRK2	leucine-rich repeat kinase 2
MMSE	Mini mental state examination
MND	motorneuron disease
MOCA	Montreal cognitive assessment
MS	multiple sclerosis
MSA	multiple system atrophy
MSSS	multiple sclerosis severity score
NfL	neurofilaments light chain
PDD	Parkinson’s disease dementia
PIGD	postural instability gait disturbance
PNFA	progressive nonfluent aphasia
PP	primary progressive
p-Tau	phosphorylated Tau
RR	relapsing–remitting
sALS	sporadic ALS
SD	semantic dementia
SIMOA	high sensitive single molecule assay
SN	substantia nigra
SP	secondary progressive
TD	tremor dominant
TDP-43	transactive DNA binding protein of ~43 kDa
t-Tau	total Tau
UPDRS	Unified Parkinson’s disease rating scale
α-syn	α-synuclein
